# Validation of maternal report of early childhood caries status in Ile-Ife, Nigeria

**DOI:** 10.1186/s12903-020-01288-z

**Published:** 2020-11-25

**Authors:** Morenike Oluwatoyin Folayan, Peter Alimi, Micheal O. Alade, Maha El Tantawi, Abiola A. Adeniyi, Tracy L. Finlayson

**Affiliations:** 1 Department of Child Dental Health, Obafemi Awolowo University, Ile-Ife, Nigeria; 2 Faculty of Dentistry, Obafemi Awolowo University, Ile-Ife, Nigeria; 3 Department of Child Dental Health, Obafemi Awolowo University Teaching Hospitals Complex, Ile-Ife, Nigeria; 4 Department of Pediatric Dentistry and Dental Public Health, Faculty of Dentistry, Alexandria University, Alexandria, Egypt; 5 Department of Preventive Dentistry, Lagos State University College of Medicine, Lagos, Nigeria; 6 San Diego State University, San Diego, USA

**Keywords:** Early childhood caries, Dental caries, Sensitivity and specificity, Predictive value of tests, Absolute and relative bias, Decision-making

## Abstract

**Background:**

To determine the validity of maternal reports of the presence of early childhood caries (ECC), and to identify maternal variables that increase the accuracy of the reports.

**Methods:**

This secondary data analysis included 1155 mother–child dyads, recruited through a multi-stage sampling household approach in Ile-Ife Nigeria. Survey data included maternal characteristics (age, monthly income, decision-making ability) and maternal perception about whether or not her child (age 6 months to 5 years old) had ECC. Presence of ECC was clinically determined using the dmft index. Maternally reported and clinically determined ECC presence were compared using a chi-squared test. McNemar's test was used to assess the similarity of maternal and clinical reports of ECC. Sensitivity, specificity, positive and negative predictive values, absolute bias, relative bias and inflation factor were calculated. Statistical significance was determined at *p* < 0.05.

**Results:**

The clinically-determined ECC prevalence was 4.6% (95% Confidence interval [CI]: 3.5–5.0) while the maternal-reported ECC prevalence was 3.4% (CI 2.4–4.6). Maternal reports underestimated the prevalence of ECC by 26.1% in comparison to the clinical evaluation. The results indicate low sensitivity (9.43%; CI 3.13–20.70) but high specificity (96.9%; CI 95.7–97.9). The positive predictive value was 12.8% (CI 4.3–27.4) while the negative predictive value was 95.7% (CI 94.3–96.8). The inflation factor for maternally reported ECC was 1.4. Sensitivity (50.0%; CI 6.8–93.2) and positive predictive value were highest (33.3%; CI 4.3–77.7) when the child had a history of visiting the dental clinic.

**Conclusions:**

Mothers under-reported the presence of ECC in their children in this study population. The low sensitivity and positive predictive values of maternal report of ECC indicates that maternal reporting of presence of ECC may not be used as a valid tool to measure ECC in public health surveys. The high specificity and negative predictive values indicate that their report is a good measure of the absence of ECC in the study population. Child’s history of dental service utilization may be a proxy measure of presence of ECC.

## Background

The gold standard for the diagnosis of oral conditions is clinical oral examination by trained dentists [1]. Yet dentists’ involvement in national oral health surveys come with huge costs resulting from purchase of materials, time of specialized personnel, fatigue of examiners, and increased probability of refusal for the examination, which would reduce the response rate [2]. Alternatively, using a questionnaire administered by an interviewer demands less time and resources in contrast to clinical examinations. However, self-reported assessments of oral health status may be biased and inaccurate. Therefore, studies exploring the validity of a range of self-reported oral health and related measures with different population groups are needed.

Population-based health surveys are increasingly utilizing self-reported health measures to obtain information about disease prevalence [3]. They have been used to measure the prevalence of cancer and cardiovascular diseases [4], juvenile rheumatoid arthritis [5], general health [6–10] and oral health [11, 12]. Self-reported measures were also adopted for obtaining information on the oral health of adults in Australia [13] and the United Kingdom [14]. These self-measures have been used among ethnically and age-diverse populations [15, 16]. A few studies also used maternal reports of children’s oral health status to assess children’s oral health status [17, 18].

Studies on the validation of self-reported oral health information have indicated low agreement regarding the decayed component of the decayed, missing and filled tooth (dmft/DMFT) index [2]. This may be related to poor recognition of dental caries by respondents [2, 19], the perception of caries only when the lesion affects their social relations, or the consciousness of caries only when they experience pain [20]. The agreement in self-reported number of missing and filled teeth and clinical findings is stronger than it is for decayed tooth [21]. Clinic attendance for tooth extraction and filling provide patients the opportunity to learn about their oral diseases thereby increasing their ability to remember their disease profile when self-reporting [2, 19]. However, agreement about extracted teeth is better among adolescents than among adults and the elderly. This may be because of the long period of time since the occurrence of tooth loss for elders in particular, making accurate recollection of the event more difficult [22]. Also, there is better correlation between clinically determined and self-reported dental caries measured by the decay, missing and filled teeth (DMFT) index than that measured by International Caries Detection and Assessment System. This may be because the DMFT index identifies advanced stages of tooth decay, which individuals can readily perceive than the early stages of caries detected by the International Caries Detection and Assessment System [22].

Studies on the sensitivity, specificity and positive predictive values of self-reported measures of oral diseases and disorders from different parts of the world are needed because the measures are influenced by personal beliefs, cultural background, and social, educational, and environmental factors [21, 23, 24]. Culture influences the way oral health and illness are perceived, symptoms are interpreted, and dental care is sought [25]. It is therefore important to identify simple and accurate self-report oral health measure that can be used in different settings to measure similar oral health phenomena [26]. The efficacy of such an inexpensive and practical tool is very relevant for use in resource-poor settings where more expensive and complicated clinical examinations are not affordable.

Although caries is a major oral health problem in children in Nigeria, there are no studies on the correlation between self-reported oral health status and clinical assessment of these lesions in Nigeria [27]. No national oral health survey has been conducted in Nigeria due to costs associated with such an effort. Yet, without such data, planning for the oral health of children in Nigeria becomes a challenge. This study will attempt to address this gap. Specifically, the objective of this study was to determine the validity of maternal reports of the presence of ECC in preschool-aged children, and to identify the variables that increase the accuracy of such reports. We hypothesize that mothers with more ability to make autonomous decisions are able to accurately report the presence of ECC in their children.

## Methods

This was a secondary analysis of a dataset of 1549 maternal-child dyads primarily collected to determine the prevalence of ECC and examine maternal psychosocial factors that were risk indicators for ECC. The data were collected from residents in Ife Central local government area, one of the 774 local government areas in Nigeria.

### Sample size

The sample size required for this secondary analysis was determined based on an assumption that 50% of mothers with autonomous decision-making ability were able to accurately report the presence of ECC in their children in the absence of any accessible data. We estimated, based on a margin of error of 5% and 95% confidence level, that the required sample size for this study was 768. We had access to data from 1155 mother–child dyads who reported the presence of ECC in their children.

### Sampling and study procedure for the primary study

A multi-stage sampling technique was used to collect the primary data. First, 70 of the 700 enumeration areas in the local government area was selected by simple random method (balloting), followed by selection of every other household on each street in the enumeration areas. The sample of the enumeration areas was limited to 10% in line with prior suggestions that a 10% sample was an adequate sample size for household surveys [28]. The final stage was the selection of an eligible respondent in each household for interview and clinical examination. Only one child and mother dyad in each household was eligible for participation in the study. A child was eligible for study participation if (s)he was below the age of 6 years, living with a caregiver, present at the time of the survey and for whom parental consent for study participation was obtained. Children with chronic medical conditions that required prolonged use of sweetened medications and those with medical conditions that increased their risk for ECC were excluded from the primary study. Trained and experienced field workers collected the data electronically using an interviewer-administered structured questionnaire on Open Data Kit—an online/offline platform for the collection and management of data. The questionnaire was administered to the mothers. Folayan et al. [29, 30] provided detailed descriptions of the sampling process and study procedure.

### Data collection

*Socio-demographics*: Information on the socio-demographic profile of the mother (age, income, educational status) were extracted from the primary study dataset. The mother’s age at her last birthday was categorized into three groups: ≤ 29 years, 30–39 years, 40 years and over. Mother’s income was defined as monthly salary for persons in paid employment, and an estimate of monthly income for self-employed persons. Income was categorized using the national Nigerian currency and wage into three categories: ≤ N18,000 ($49)/month, N18,001–N60,000/month, and > N60,000 (168)/month [31]. Mothers’ educational status was defined as no formal education, primary school only, secondary school only, or tertiary (post-secondary) education.

#### Women decision-making ability

Data about women’s participation in making decisions concerning (1) their own health care, (2) major household purchases, and (3) visits to family or relatives without having to get permission were extracted from the primary study dataset. The questions exploring women’s decision-making ability were adopted from the Nigeria Demographic and Health Survey [32]. When others make any of these decisions on behalf of the mother, the mother was regarded as having no decision-making ability for the item scored.

#### Child dental visit history and ECC status

Data on children’s dental visit history were extracted from the primary study dataset. Mothers were asked if their children had ever visited the dentist (yes or no), and if the child had a hole in their teeth (yes or no). The prevalence of ECC was determined as the proportion of children reported by their mothers to have caries.

#### Oral examination

Data on the early childhood caries profile of the 1155 children generated in the primary study by five calibrated dentists who conducted the oral examination for each child were extracted for this study. Calibration of the five dentists was conducted by first training them on caries assessment using a colored picture chart with varying presentations of decayed, missing and filled teeth, followed by examining a group of five children with caries and making a diagnosis using the World Health Organization scoring criteria. The scoring for each of the five children was repeated three times with an interval of one week between each visit. Intra-examiner agreement for each of the dentists was calculated using Cohen’s Kappa and the inter-examiner agreement (between the dentists and the trainer) was calculated using the Cohen’s kappa coefficient. The intra- and inter-examiner reliability tests were all greater than 0.80.

ECC was determined in the primary study using the decayed-missing-filled teeth (dmft) index as recommended by the World Health Organization [33]. Radiographic assessment was not conducted. The dmft score was an aggregated score of the d, m and f scores for each child. ECC was considered present when the dmft score was > 0 and absent when the dmft was 0. The study had access to the aggregated dmft score for each child and not the respective d, m and f scores.

### Data analysis

The final analytic sample included only children who had maternally reported and clinically determined presence of ECC (N = 1155). Descriptive analyses were performed, including calculation of mean values and 95% confidence intervals (CI) for maternal reported and clinically determined presence of ECC.

The bivariate association was tested between clinical and maternal reported ECC, and selected maternal characteristics separately using a chi-squared test. McNemar's test was used to assess differences between paired data (i.e. clinical versus maternal reporting of ECC).

In addition, sensitivity, specificity, positive and negative predictive values, absolute bias, relative bias and inflation factor (gold standard prevalence/self-reported prevalence) were also calculated [34]. Estimates of sensitivity, specificity, and positive and negative predictive values were stratified by the socio-demographic profile of the mother (age, income, educational status) and maternal decision-making status. Statistical analyses were conducted with Intercooled STATA (release 15) for windows. Statistical significance was inferred at *p* ≤ 0.05.

### Ethics approval

Ethical approval for the study was obtained from the Obafemi Awolowo University Teaching Hospitals Complex Health Research Ethics Committee (NHREC/27/01/2009a and IRB/EC/0004553). Study participants for the primary study were recruited after receiving written consent from the mothers for their own study participation, and written consent for their child’s participation in the study.

## Results

Table 1 shows the profile of mother–child dyads in the study. There were missing data for the variables on maternal decision-making status concerning (1) their own health care (n = 19), (2) major household purchases (n = 24), and (3) visits to family or relatives without having to take permission (n = 32). There were no statistically significant differences in the proportion of children with clinically determined ECC by maternal age, schooling, maternal income, decision-making status and child’s history of dental services utilization (n = 21).

**Table 1:** Relationship between maternal socio-demographic profile and clinically assessed and mother-reported presence of early childhood caries

Variables	Clinical evaluation of early childhood caries					Maternal reporting of early childhood caries					*p* value for McNemar test^a^
	Absent		Present		*P* value	Absent		Present		*P* value	
	Number	%	Number	%		Number	%	Number	%		
Age of mother											
< 29 yrs	384	34.8	13	24.5	0.251^b^	383	96.5	14	3.5	0.845^b^	1.000
30–39 yrs	602	54.6	35	66.0		615	96.5	22	3.5		0.085
> 40 yrs	116	10.5	5	9.4		118	97.5	3	2.5		0.727
Mother’s income											
≤ N18,000 ($49.00)/month	307	27.9	19	35.8	0.217	320	98.2	6	1.8	< 0.001	0.011
N18,001–N60,000/month	450	40.8	23	43.4		467	98.7	6	1.3		0.002
> N60,000 ($168.00)/month	345	31.3	11	20.8		329	92.4	27	7.6		0.007
Mother’s educational status											
No formal/primary education	96	8.7	8	15.1	0.286^b^	100	96.2	4	3.8	0.169^b^	0.344
Secondary	715	64.9	32	60.4		727	97.3	20	2.7		0.104
Tertiary	291	26.4	13	24.5		289	95.1	15	4.9		0.845
Mother’s ability to access health care for herself independently											
No	796	73.4	37	71.2	0.717	825	99.0	8	1.0	0.001	< 0.001
Yes	288	26.6	15	28.8		272	89.8	31	10.2		0.017
Mother’s ability to make household purchase independently											
No	827	76.5	38	76.0	0.935	857	99.1	8	0.9	< 0.001	< 0.001
Yes	254	23.5	12	24.0		235	88.3	31	11.7		0.003
Mother’s ability to make family visits independently											
No	806	75.3	39	75.0	0.967	837	99.1	8	0.9	< 0.001	< 0.001
Yes	265	24.7	13	25.0		248	89.2	30	10.8		0.008
Child history of dental service utilization											
Never used	1031	95.3	48	92.3	0.313^b^	1049	97.2	30	2.8	0.001	0.047
Ever used	51	4.7	4	7.7		49	89.1	6	10.9		0.687

^a^McNemar's test on paired data of clinical versus maternal evaluation of caries for N = 1155 children with both clinical and maternal evaluation of ECC

^b^Fisher exact test compute

There were statistically significant differences in the proportion of children with maternal-reported ECC by income, (*p* < 0.001) maternal decision-making status (*p* < 0.001) and child’s history of dental service utilization (*p* = 0.001). The proportion of children reported to have ECC was significantly higher for mothers with the highest income, mothers who could make more independent decisions, and those who had utilized dental services before.

There were also statistically significant underestimations of the proportion of children with clinical ECC by mothers with income < 18,000 (49$)/month (*p* = 0.011), mothers with no ability to access health care for herself independently (*p* < 0.001), mothers who cannot make household purchases independently (*p* < 0.001), and mothers who cannot make family visits independently (*p* < 0.001). Additionally, mothers with children who had never visited a dentist underestimated the presence of ECC in their child (*p* < 0.001).

There were statistically significant overestimations of the presence of ECC by mothers with income > 60,000 (168$) (*p* = 0.007), those with ability to access health care for herself independently (*p* = 0.017), mothers who can make household purchases independently (*p* = 0.003), mothers who can make family visits independently (*p* = 0.008), and mothers who could make all three decisions (*p* = 0.014).

Table 2 is a description of the agreement between clinically determined and maternal reported ECC prevalence of the study cohort. The clinical prevalence of ECC for the study cohort was 4.6 (95% CI 3.5–5.0)% while the maternal-reported ECC prevalence was 3.4 (95% CI 2.4–4.6)%. Though there was no statistically significant difference in the clinically determined and maternal reported ECC prevalence (*p* = 0.136), maternal reports underestimated the prevalence of ECC by 26.1% in comparison to the clinical evaluation. The results indicate low sensitivity (9.43%) but high specificity (96.90%). The positive predictive value of 12.8% indicates that among those identified with ECC, a minority actually had the condition. The negative predictive value indicates that among those identified as not having ECC, 95.7% were confirmed as actually not having the condition. The inflation factor for maternally reported presence of ECC was 1.4.

**Table 2:** Prevalence of clinically-determined and maternal-reported ECC with estimates of sensitivity, specificity, positive predictive value, negative predictive value, absolute bias, relative bias and inflation factor of children 0–5 years old, Ile-Ife, Nigeria 2018 (N = 1155)

	Clinically assessed	Maternally reported
Prevalence (95% CI)	4.6 (3.5–5.0)	3.4 (2.4–4.6)
*P* value of McNemar test	0.151	
Sensitivity% (95% CI)	9.43 (3.1–20.7)	
Specificity % (95% CI)	96.9 (95.7–97.9)	
Positive predictive value % (95% CI)	12.8 (4.3–27.4)	
Negative predictive value % (95% CI)	95.7 (94.3–96.8)	
Absolute bias	− 1.2	
Relative bias	26.1	
Inflation factor	1.4	

Absolute bias = tested prevalence − gold standard (clinical assessment) prevalence

Relative bias = absolute bias/gold standard (clinical assessment) prevalence × 100

Inflation factor = gold standard (clinical assessment) prevalence/tested (maternal assessment) prevalence

Table 3 shows the estimates of sensitivity, specificity, positive and negative predictive values. Sensitivity was higher when the child had a history of visiting the dental clinic (50.0%; CI 6.8–93.2); mothers 30–39 years (11.4%; CI 3.2–26.7). Also, sensitivity was highest for mothers who had the highest income (27.3%; CI 6.0–61.0); and higher for mothers who could take decisions on health access (20.0%; CI 4.3–48.1), household purchases (25.0%; CI 5.5–57.2) and family visits (23.1%; CI 5.0–53.8).

**Table 3:** Sensitivity, specificity, positive and negative predictive values for clinically determined and maternally-reported ECC according to maternal schooling, household income, empowerment status and child’s visit to dentist in previous year in children 0–5 years old, Ile-Ife, Nigeria

	Sensitivity	Specificity	Positive predictive value	Negative predictive value
Child history of dental service utilization				
Never used	4.2 (0.51–14.3)	97.3 (96.1–98.2)	6.7 (0.82–22.1)	95.6 (94.2–96.8)
Ever used	50.0 (6.8–93.2)	92.2 (81.1–97.8)	33.3 (4.3–77.7)	95.9 (86–99.5)
Mothers’ education level				
Primary/No formal education	12.5 (0.3–52.7)	96.9 (91.1–99.4)	25.0 (0.6–80.6)	93.0 (86.1–97.1)
Secondary	9.4 (2.0–25.0)	97.6 (96.2–98.6)	15.0 (3.2–37.9)	96.0 (94.3–97.3)
Tertiary	7.9 (0.2–36.0)	95.2 (92.1–97.3)	6.7 (0.2–31.9)	95.8 (92.9–97.8)
Mothers’ age				
< 30 years	7.7 (0.2–36.0)	96.6 (94.3–98.2)	7.14 (0.18–33.9)	96.9 (94.6–98.4)
30–39 years	11.4 (3.2–26.7)	97.0 (95.3–98.2)	18.2 (5.2–40.3)	95.0 (92.9–96.5)
≥ 40 years	0.0 (0.0–52.2)	97.4 (92.6–99.5)	0.0 (0.0–70.8)	95.8 (90.4–98.6)
Mother’s income				
≤ N18,000 per month	5.3 (0.1–26.0)	98.4 (96.2–99.5)	16.7 (0.4–64.1)	94.4 (91.3–96.6)
N18,001–N60,000 per month	4.4 (0.1–21.9)	98.9 (97.4–99.6)	16.7 (0.4–64.1)	95.3 (93.0–97.0)
> N60,000 per month	27.3 (6.0–61.0)	93.0 (89.8–95.5)	11.1 (2.4–29.2)	97.6 (95.3–98.9)
Mother’s ability to access health care for herself independently				
No	5.4 (0.7–18.2)	99.2 (98.4–99.7)	25.0 (3.2–65.1)	95.8 (94.1–97.0)
Yes	20.0 (4.3–48.1)	90.3 (86.3–93.4)	9.7 (2.0–25.8)	95.6 (92.4–97.7)
Mother’s ability to make household purchase independently				
No	5.3 (0.6–17.7)	99.3 (98.4–99.7)	25.0 (3.2–65.1)	95.8 (94.2–97.0)
Yes	25.0 (5.5–57.2)	89.0 (84.5–92.5)	9.7 (2.0–25.8)	96.2 (92.9–98.2)
Mother’s ability to make family visits independently				
No	5.1 (0.6–17.3)	99.3 (98.4–99.7)	25.0 (3.2–65.1)	95.6 (94.0–96.9)
Yes	23.1 (5.0–53.8)	89.8 (85.5–93.2)	10.0 (2.1–26.5)	96.0 (92.7–98.0)

The positive predictive value was higher when the child had a history of visiting the dental clinic (33.3%; CI 4.3–77.7); and mothers between 30 and 39 years old (18.2%; CI 5.2–40.3). It was also lowest for mothers with the highest income (11.1%; CI 2.4–29.2), and mothers who could not make decisions on health access (25.0%; CI 3.2–65.1), household purchases (25.0%; CI 3.2–65.1) and family visits (25.0%; CI 3.2–65.1). Specificity and negative predictive values were > 90% for all subgroups.

## Discussion

The study findings indicated that maternal-reported ECC presence exhibited low sensitivity but high specificity. Mothers who were empowered (as demonstrated by their ability to make decisions about their health access, household purchases and family visits) had better sensitivity of their report of their child’s ECC status, though their positive predictive value was low. The report on ECC presence by mothers of children who had a history of dental service utilization had the highest level of sensitivity. Our study hypothesis was, therefore, partially nullified.

The findings of this study suggest that maternal report of ECC are largely influenced by social-economic factors and maternal decision-making ability. Prior studies also indicated that maternal decision-making ability has significant impact on the health status of her child [35–37]: the ability to socialize improves access to oral health information, which may improve awareness of the oral health status of the child. For this reason, mothers with better socio-economic status and decision-making ability may be more likely to use oral health facilities [38] where they get to learn about their children’s ECC status.

This study also highlights that young children’s history of dental service utilization was associated with the highest sensitivity of maternally reported presence of ECC. Dental visits, particularly early in life, create an opportunity for caregivers to be educated about the oral health status of children. Studies in Nigeria indicate that the majority of children use dental services for curative rather than preventive reasons [39]. It is likely that children with a history of dental service utilization had visited the dentist for pain management and thus, their primary caregiver (who often is the mother), would have been informed about the presence of caries lesion in the child’s mouth. This may explain the highest sensitivity of maternal report of ECC status of their children in this subgroup.

Mothers who were empowered were more likely to overestimate presence of ECC. The over-reporting of ECC by mothers does not seem to be related to a social desirability bias, wherein mothers report what the expected societal norm should be [40]. Rather, this over-reporting may be the report of internalized societal, community, or group norms as socially expected oral health status of the child. Mothers with high income and high decision-making ability are likely to have better understanding of what is socially expected regarding caries status of very young children: they are aware of the high caries risk of children associated with high sugar consumption. In a transiting economy like Nigeria, children with high socioeconomic status are more at risk for caries [41]. Those mothers then pragmatically interpret the question to mean expected social oral health identity of children and responds to this interpretation rather than the semantic meaning of the question [42].

These research findings have oral health policy implications. Nigeria has no national oral health survey to determine the prevalence, burden, and severity of ECC. Population surveys require huge outlays of financial resources. It may be implied that resource-poor communities like Nigeria cannot afford to conduct oral health surveys, or may conduct them sporadically because of inadequate funding for oral health [43]. The only national oral health survey in Nigeria was conducted in 1995 and the target populations were adolescents, young persons and adults [44, 45]. If cost-saving measures are to be taken, maternal-reported measures of young children’s caries status can be done. While the overall maternal reported prevalence in Nigeria will be underestimated, the difference between the maternally reported and clinically determined prevalence may likely be minimal. The routinely implemented national demographic health survey (conducted every five years) provides an opportunity to integrate oral health questions as a way of determining the national ECC prevalence at regular intervals. This can be an efficient approach to monitoring oral health in a difficult to reach group like preschoolers in a resource-constrained setting where other priorities compete for resources. However, it has to be emphasized that maternally reported prevalence will underestimate the true prevalence of ECC.

If mothers' reporting of ECC in their children is used to plan oral health care programs, it is also important to incorporate the accuracy profile of this method in the healthcare system workflow. The high specificity and negative predictive values of maternal reporting of ECC can be used to exclude children without ECC from further contact with the healthcare system. It is, however, not advisable to refer children for further care based on maternal reporting of ECC presence due to the low sensitivity and positive predictive values of such reports as indicated in the study findings. Also, using maternal report of ECC to screen out children from receiving curative caries care may be of limited value since all young children are required to have access to preventive oral health care.

The study has a few limitations. First, we only had access to the aggregated dmft score. We could not extract the d scores for each child, as the data we had access to provided a dmft score and not the independent component scores. The d score is more appropriate for comparing maternal reporting of ECC presence. However, previous research has shown that the bulk of the dmft in preschool children in Nigeria is formed by the d component [46]. Second, this study was conducted in only one of the 774 local government areas in Nigeria. It is therefore difficult to generalize the findings to Nigeria, as there are local governments with higher proportions of women with high income and maternal empowerment status; and higher proportion of children with history of dental service utilization Also, the wide confidence interval indicates that the sample size for children with ECC was small, and a repeat study with a large population of children with ECC may produce more precise predictive values. Despite these limitations, this study contributes new information. It is the first to report on the validity of maternal reporting of ECC to determine the caries status of children under 6 years of age. It is also the first to report on the validity of using self-report to determine ECC status in a resource-limited setting. Future studies are needed to build on the study findings one of which should explore the effect of maternal oral health literacy on the accuracy of maternal reporting. We were unable to explore this in the current study due to the unavailability of this data in the primary study.

## Conclusion

Mothers under-reported the presence of ECC in their children in this study population. The low sensitivity and positive predictive values of maternal report of ECC indicates that maternal reporting of presence of ECC may not be used as a valid tool to measure ECC in public health surveys. However, the high specificity and negative predictive values indicate that their report is a very good measure of the absence of ECC in the study population. Maternal report of children’s history of dental service utilization may be a good proxy measure of presence of ECC.

## Data Availability

The data used for this study are presented in the study. The primary study from which data was extracted for this study is not yet published. Data can however be accessible on request.
